# Threshold uncertainty, early warning signals and the prevention of dangerous climate change

**DOI:** 10.1098/rsos.240425

**Published:** 2025-03-21

**Authors:** Mark J. Hurlstone, Ben White, Ben R. Newell

**Affiliations:** ^1^ Department of Psychology, Lancaster University, Lancaster LA1 4YW, UK; ^2^ School of Psychological Science, University of Western Australia, Perth, Western Australia 6039, Australia; ^3^ School of Agriculture and Environment, University of Western Australia, Perth, Western Australia 6039, Australia; ^4^ School of Psychology, UNSW, Sydney, New South Wales 2052, Australia; ^5^ Institute for Climate Risk & Response, UNSW, Sydney, New South Wales 2052, Australia

**Keywords:** cooperation, dangerous climate change, early warning signals, threshold uncertainty

## Abstract

The goal of the Paris Agreement is to keep global temperature rise well below 2°C. In this agreement—and its antecedents negotiated in Copenhagen and Cancun—the fear of crossing a dangerous climate threshold is supposed to serve as the catalyst for cooperation among countries. However, there are deep uncertainties about the location of the threshold for dangerous climate change, and recent evidence indicates this threshold uncertainty is a major impediment to collective action. Early warning signals of approaching climate thresholds are a potential remedy to this threshold uncertainty problem, and initial experimental evidence suggests such early detection systems may improve the prospects of cooperation. Here, we provide a direct experimental assessment of this early warning signal hypothesis. Using a catastrophe avoidance game, we show that large initial—and subsequently unreduced—threshold uncertainty undermines cooperation, consistent with earlier studies. An early warning signal that reduced uncertainty to within 10% (but not 30%) of the threshold value catalysed cooperation and reduced the probability of catastrophe occurring, albeit not reliably so. Our findings suggest early warning signals can trigger action to avoid a dangerous threshold, but additional mechanisms may be required to foster the cooperation needed to ensure the threshold is not breached.

## 1. Introduction

The goal of the United Nations Framework Convention on Climate Change (UNFCCC) is to achieve ‘stabilization of greenhouse gas concentrations in the atmosphere at a level that would prevent dangerous anthropogenic interference with the climate system’ [1]. But what constitutes dangerous interference? In 2009, the signatories of the Copenhagen Accord reached an agreed definition, namely that in accordance with ‘the scientific view the increase in global temperature should be below 2 degrees Celsius’ [2]. It is the fear of crossing this dangerous threshold that provides the free-rider deterrent in contemporary climate agreements. The effectiveness of this deterrent depends upon its credibility, specifically the credibility of the science of locating the critical threshold [3].

However, there is no scientific view that 2°C is the threshold for dangerous anthropogenic interference. Although there is a consensus regarding the existence of dangerous climate thresholds, the location of those thresholds is highly uncertain and the subject of considerable scientific debate [4–6]. For example, based on the goal of preserving the large polar ice sheets, Rockstrom *et al*. [6] identify a ‘planetary boundary’ of atmospheric carbon dioxide concentration of somewhere between 350 and 550 parts per million by volume (a boundary that has already been exceeded). However, the location of the critical threshold within this boundary that could trigger the abrupt collapse of the ice sheets is unknown.

Political actors and climate negotiators are not oblivious to this scientific uncertainty. No sooner had the signatories of the Copenhagen Accord agreed upon the 2°C target than a year later in Cancun, discussions were raised regarding the possibility of adopting a 1.5°C target. This uncertainty is enshrined in the Paris Agreement, which—in addition to reaffirming the 2°C target—underscores the desirability of ‘pursuing efforts to limit the temperature increase to 1.5°C’ [7].

### 1.1. Threshold uncertainty and collective action

What are the consequences for the climate negotiations of uncertainty about climate thresholds? Recently, an experimental literature has emerged to tackle this question. Within this literature, the problem of avoiding dangerous climate change has been simulated using laboratory cooperation experiments (for reviews, see [8–10]). In these experiments, groups of players must cooperate by investing money from a personal operating fund into hypothetical emission abatement to avoid crossing a dangerous threshold, which, if breached, triggers catastrophic economic losses for all. This literature finds that when the threshold is known with certainty, groups can effectively coordinate their efforts to remain on the safe side of the dangerous threshold, but when the threshold is uncertain, coordination collapses, and catastrophe is all but guaranteed [11–14]. Although threshold uncertainty impedes cooperation compared to when the threshold is known with certainty, it nevertheless facilitates cooperation compared to when there is no threshold at all [15]. This suggests the framing of the climate negotiations in terms of avoiding ‘dangerous’ instead of ‘gradual’ climate change has been beneficial [15]—faced with an uncertain threshold, countries may reduce their emissions more than if they were unaware of a threshold for dangerous climate change. However, it may not be enough to prevent countries from crossing the dangerous threshold.

An additional feature of these and other threshold experiments is that under threshold certainty, there is a strong alignment between what groups propose to contribute, pledge to contribute and actually contribute, whereas under threshold uncertainty, pledges tend to be lower than proposals, and actual contributions fall short of pledges [12,14–16]. The parallels with the real climate negotiations are striking and sobering. Under the Paris Agreement, countries have proposed to do less than is required to limit the risk of catastrophe (the agreement aims to restrict warming to 2${,}^{\circ }$C but recognizes that a 1.5${,}^{\circ }$C goal is probably required) and pledged to contribute less than is required to reach the collective goal [7,17,18]. Laboratory cooperation experiments suggest countries’ actual contributions will be less than their pledges, leaving little hope of staying below the 2°C limit [16].

A clear implication of the results of threshold experiments is that if climate scientists could reduce the uncertainty surrounding the location of the dangerous threshold sufficiently, then this might provide the leverage necessary to transform the climate negotiations. Uncertainty about the location of a dangerous threshold can be reduced through the detection of early warning signals of approaching climate transitions [19–23]. For example, strong positive feedback in the internal dynamics of the climate system or generic statistical indicators of loss of system resilience could provide indications that a climate tipping point is approaching [23].

That such early warning signals might facilitate cooperation was demonstrated in an experiment by Barrett & Dannenberg [14] that parametrically varied the degree of uncertainty surrounding the threshold. In their experiment, participants were randomly allocated to groups of 10 players. Each player was given €31, which was divided into an operating fund of €11 and an endowment of €20. The operating fund could be used to invest in ‘weak’ or ‘strong’ abatement by purchasing poker chips (max = 10 of each type) at a cost of €0.10 or €1.00, respectively. The game was played over a single round divided into the following two stages: a communication stage, where each player submitted a proposal regarding the contribution target for the group and pledged an amount they would contribute individually (both proposals and pledges were non-binding), followed by a contribution stage where each player chose how many poker chips they would actually contribute. Players received €0.05 for each poker chip contributed by the group, regardless of its cost. Critically, if the total number of poker chips contributed by the group was less than a threshold value, then €15 was deducted from each player’s endowment, which represented the impact (i.e. damages) of failing to reach the threshold.

The experiment comprised five treatments, each containing 10 groups. In the certainty treatment, the threshold was 150, whereas in four threshold-uncertainty treatments, it was a uniformly distributed random variable between either 100−200 (100% uncertainty), 135−165 (30% uncertainty), 140−160 (20% uncertainty) or 145−155 (10% uncertainty).

The results revealed the sensitivity of collective action to the degree of uncertainty about the tipping point. When the threshold was certain, 80% of groups avoided catastrophe, whereas this value plummeted to 0% in treatments 100−200, 135−165 and 140−160, where the degree of threshold uncertainty varied between 100% and 30%. However, in treatment 145−155, where threshold uncertainty was reduced to within 10% of the threshold value, 40% of groups avoided catastrophe.

The results of Barrett & Dannenberg [14] suggest early warning signals that reduce uncertainty about the proximity of a dangerous climate threshold might catalyse action to avoid it, provided that uncertainty is reduced to within a very narrow range. However, there are two potential limitations of this study. First, it employed a one-shot game that fails to capture the repeated nature of the real game of climate change in which countries interact continuously and one country’s decision about how much to abate is informed by how much other countries have pledged to abate, how much they have actually abated and the consistency between stated intentions and behaviour. However, in the one-shot game, beliefs about how much others will abate can only be informed by others’ pledges, not actual abatements. Second, groups in the uncertainty treatments were always confronted with the same level of threshold uncertainty (threshold uncertainty varied between but not within treatments). However, in the real climate game, an early warning signal would arrive against the backdrop of initial threshold uncertainty. Thus, a more realistic assessment of the early warning signal hypothesis requires an experimental scenario wherein groups face threshold uncertainty initially, followed by a reduction in that uncertainty as the threshold is approached. Under this scenario, we might expect an early warning signal to be less effective at catalysing cooperation. For example, the relatively large threshold uncertainty faced by groups initially might cause cooperation to collapse to a point from which recovery is difficult, given the remaining time available.

### 1.2. Coordination devices and equilibria

In the current paper, we present an experiment designed to address these important issues. In doing so, our experiment allows us to address a theoretical question that has hitherto largely been ignored in this literature: if a group of players start by coordinating around one equilibrium, can they subsequently be shifted to another via some coordination device—a mechanism that coordinates the activities of individuals to prevent coordination failures—be it an early warning system or some other instrument.

At least two previous studies have presented results that bear on this question. In a study by Tavoni *et al*. [24], groups of six players undertook 10 rounds of a climate cooperation game with a certain threshold. In rounds 1−3, the software determined contributions such that three poor players were forced to contribute the maximum possible per round, whereas three rich players were forced to contribute nothing. In rounds 4−10, players could choose how much to contribute. In this situation, groups became locked into the pattern of contributions set initially by the software—that is, the rich players continued to contribute much less than the poor players. However, in another treatment, Tavoni *et al*. [24] introduced a coordination device—on rounds 4 and 7, players could submit non-binding pledges regarding how much they intended to contribute by the end of the game. Communication greatly increased the probability of avoiding catastrophe. This was because the rich players were able to signal to the poor players their willingness to compensate for their lesser resource capacity and the poor players were willing to trust that the rich players would honour their pledges. Communication therefore moved the groups to a new equilibrium compared to when this coordination device was unavailable.

In another study, Milinski *et al*. [25] had six-player groups undertake a 10-round climate cooperation game with a certain threshold. They examined whether another form of coordination device, namely an intermediate threshold that must be reached by the middle of the game, would increase the probability of avoiding crossing the final threshold. Without an intermediate threshold, contributions were relatively stable over rounds, whereas with an intermediate threshold, contributions rose towards a peak mid-game, before dropping sharply and then rising again. Thus, the presence of an intermediate threshold altered the dynamics of contributions and moved groups towards a new mid-game equilibrium. However, and critically, the intermediate threshold also modestly increased the probability of groups reaching the equilibrium at the end of the game needed to avoid crossing the final threshold, compared to the situation without an intermediate threshold.

In summary, there is some evidence from iterated threshold experiments with a certain threshold that coordination devices based on communication and intermediate thresholds can encourage groups to coordinate on a new equilibrium [24] or coordinate on one equilibrium and increase the probability of then coordinating on another [25]. In the current study, we address this issue in the context of an iterated threshold experiment involving threshold uncertainty and early warning signals of varying precision.

### 1.3. Current research

Our experiment involved 240 participants who were allocated to six-player groups to play a catastrophe avoidance game developed by Milinski *et al*. [26] and subsequently augmented by Dannenberg *et al*. [12] to include a communication component and study threshold uncertainty effects. Each player was given a $40 endowment. In each of the 10 rounds, players decided whether to contribute $0, $2 or $4 into a catastrophe avoidance account. Players knew if the total amount contributed by the end of the game did not equal or exceed a threshold amount, they would lose 90% of their remaining endowment. Before the contribution decisions on rounds 1 and 6, each player submitted the following two non-binding communications: (i) a proposal regarding how much the group should collectively contribute over the 10 rounds, and (ii) a pledge regarding how much they personally intended to contribute toward reaching this collective goal.

The experiment involved four treatments (certainty, uncertainty, warning wide and warning narrow), each comprising 10 groups. The certainty and uncertainty treatments are identical to the certainty and risk (i.e. uncertainty) treatments from the study by Dannenberg *et al.* [12]. The threshold was certain in the certainty treatment, whereas it was uncertain in the uncertainty, warning-wide and warning-narrow treatments. In the certainty treatment, groups were told the threshold was $120, whereas, in the other treatments, they were informed it was a random amount between $0 and $240, with each whole dollar amount having an equal probability of being selected, but the exact amount would not be determined and announced until the conclusion of the game. The warning-wide and warning-narrow treatments differed from the uncertainty treatment in that in round 6—before the second set of non-binding proposals and pledges—unexpectedly, groups received an early warning signal that the uncertainty surrounding the threshold had been reduced. Specifically, in the warning-wide treatment, groups were instructed the threshold was now a random amount between $84 and $156 (reducing uncertainty to within 30% of the threshold value), whereas, in the warning-narrow treatment, they were instructed the threshold was now a random amount between $108 and $132 (reducing uncertainty to within 10% of the threshold value). Thus, the uncertainty treatments (uncertainty, warning wide and warning narrow) were all based on a uniform distribution with an expected threshold value of $120.

The structure of the rest of this paper is as follows: we begin by reporting the detailed methods of our experiment, followed by the predictions and game equilibria. We then present the experimental results before discussing their relationship to the background literature and their implications for climate negotiations.

## 2. Methods

Ethical approval to conduct the experiment was granted by the Human Ethics office at the University of Western Australia (UWA; RA/4/1/6996: Committing to the public good).

### 2.1. Participants

Two hundred and forty members of the campus community at the UWA participated in the experiment (mean age = 24.37 years; s.d. = 7.30; range = 17−56; 146 females and 93 males, 1 gender unspecified). Participants were recruited using the Online Recruitment System for Experimental Economics (ORSEE) [27], an open-source Web-based recruitment platform used by the Behavioural Economics Laboratory at UWA. The ORSEE database contains a pool of over 1500 UWA staff and students from a range of academic disciplines. Participants were recruited by issuing electronic invitations to randomly selected individuals in the ORSEE database to attend the experimental sessions.

### 2.2. Design

The experiment employed a four (treatment: certainty vs. uncertainty vs. warning wide vs. warning narrow) × 10 (round: 1−10) mixed design: treatment was a between-groups factor, whereas round was a within-groups factor. Participants were tested in groups of six players (10 groups per treatment). We commenced testing with the uncertainty treatments (uncertainty, warning wide and warning narrow)—randomly allocating each six-person group to one of the three treatments—before collecting the data for the certainty treatment. Despite the non-random allocation to the certainty treatment, there was no evidence that participants in this treatment differed significantly from those in the other treatments on the basis of age (Kruskal–Wallis, $\chi_{df=3}^{2}$ = 1.22, *p* = 0.748), gender (Kruskal–Wallis, $\chi_{df=3}^{2}$ = 1.68, *p* = 0.642) or responses on a post-game economic preferences questionnaire (see electronic supplementary material, statistical analyses). Table 1 provides a summary of the experimental design, which is elaborated below.

**Table 1.** Overview of the design of the experiment.

treatment	Q rounds 1−10		expected value	N participants
certainty	$120		$120	10 × 6 = 60
uncertainty	[$0, $240]		E( Q ) = $120	10 × 6 = 60
	Q rounds 1−5	Q rounds 6−10		
warning wide	[$0, $240]	[$84, $156]	E( Q ) = 120	10 × 6 = 60
warning narrow	[$0, $240]	[$108, $132]	E( Q ) = 120	10 × 6 = 60

Q, threshold for catastrophe.

### 2.3. Apparatus, materials and procedure

Experimental sessions were conducted in the Behavioural Economics Laboratory, a computerized laboratory for running economic experiments at UWA, in the presence of two experimenters. At the start of a session, players were randomly seated at interconnected computer terminals running the Zurich Toolbox for Readymade Economic Experiments (z-Tree) [28], which was used to register and communicate their decisions during the experiment. The computer terminals were separated by privacy blinds to prevent player collusion. Participants read an information sheet and provided written informed consent initially, after which they read the experimental instructions and answered a series of control questions (see electronic supplementary material, experimental instructions) to ensure they understood the rules of play. The experiment did not commence until the experimenters had verified that all players had answered the control questions correctly. To ensure anonymity, each player was assigned a pseudonym before the game commenced (Ananke, Telesto, Despina, Japetus, Kallisto or Metis). During the game, each player’s decisions were communicated to the other players under their designated pseudonyms.

**Figure 1.**
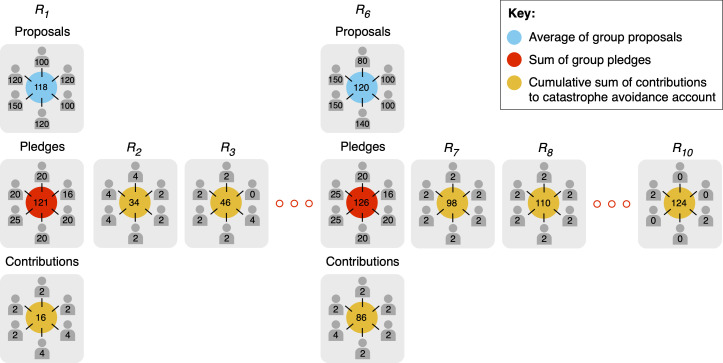
An illustration of the structure of the catastrophe avoidance game. At the start of the game, $40 is credited to the personal account of each player (N = 6). In the certainty treatment, players are instructed that the threshold is $120, whereas in the uncertainty, warning-wide and warning-narrow treatments, players are told the threshold is a uniform random value between $0 and $240, but they will not know the actual value of the threshold until the end of the game. In each of the 10 rounds, *R*${,}_{1-10}$, each player must decide simultaneously and independently whether to contribute $0, $2 or $4 from their personal account into a damage prevention account. At the start of round 1—and again in round 6—players simultaneously and independently submit two non-binding announcements before making their contribution decision. First, each player submits a ‘proposal’ regarding the target level of contributions the group should aim for by round 10, and the average of these proposals becomes the agreed collective target. Next, each player submits a ‘pledge’ regarding the total amount that they will personally contribute across the 10 rounds toward reaching the agreed collective target. In the warning-wide and warning-narrow treatments, before players submit their second set of non-binding proposals in round 6, they are instructed that the uncertainty about the threshold has reduced and that the threshold is now a uniform random value between $84 and $156 (warning wide) or $108 and $132 (warning narrow). At the end of the game, the contributions in the damage prevention account are compared with the known (certainty treatment) or randomly chosen (uncertainty, warning-wide and warning-narrow treatments) threshold. In the uncertainty treatments, the computer determines the exact threshold amount by drawing a random number from a uniform distribution either over the interval [0, 240] (uncertainty treatment), [84, 156] (warning-wide treatment) or [108, 132] (warning-narrow treatment). If the total contributions equal or exceed the threshold, then the damage is avoided, and players get to keep the remaining contents of their personal accounts; otherwise, they lose 90% of their remaining funds.

The structure of the game is depicted in figure 1. At the start of the game, each player was given a $40 endowment. In each of 10 rounds, players decided simultaneously and independently whether to contribute $0, $2 or $4 of their endowment into an account for damage prevention. Players knew that the total amount invested in the damage prevention account by the end of the game must equal or exceed a threshold amount; otherwise, each player would lose 90% of their remaining endowment. In the certainty treatment, the instructions emphasized that the threshold amount to be reached by the end of the game was $120. By contrast, in the uncertainty treatments (uncertainty, warning wide and warning narrow), the instructions emphasized that the threshold amount was a random amount between $0 and $240, with each whole dollar amount having an equal probability of being selected, but the exact amount would not be determined and declared until the conclusion of the game.

At the start of rounds 1 and 6, each player simultaneously and independently submitted two non-binding announcements. First, each player submitted a proposal regarding how much the group should contribute in total over the 10 rounds. After each player had registered their proposal, the proposals of all players, as well as the group average, were displayed on all computers simultaneously. Players knew that the average group proposal would serve as the agreed collective target. Second, each player submitted a pledge regarding how much money they would personally contribute in total over the 10 rounds. Once each player had registered their pledge, the pledges of all players, as well as the group total, were displayed on all computers simultaneously along with the group proposals to facilitate comparison.

At the end of each round, the contribution decisions of all six players, their cumulative contributions across all rounds played so far and their proposals and pledges were displayed on all computers simultaneously (in addition to the total current round contributions, total contributions across all rounds played so far, average group proposal and total group pledges). In this way, as the game progressed, players were able to gauge whether their group members were adhering to their pledges and whether the group contributions were consistent with achieving the agreed (average) group proposal.

At the start of round 6, before the second set of non-binding announcements, groups in the warning-wide and warning-narrow treatments were given an on-screen warning informing them that the uncertainty surrounding the location of the threshold had now been reduced. Specifically, in the warning-wide treatment, groups were informed that the threshold amount was now a random amount between $84 and $156 (equivalent to a 70% reduction in threshold uncertainty), whereas, in the warning-narrow treatment, groups were informed that the threshold amount was now a random amount between $108 and $132 (equivalent to a 90% reduction in threshold uncertainty). In the certainty and uncertainty treatments, the known threshold ($120) and uncertain threshold range ($0–$240), respectively, remained the same as specified at the outset, and groups in these treatments did not, therefore, receive any additional information about the threshold. Instead, at the start of round 6, groups in these treatments proceeded directly to submit their second set of non-binding announcements.

At the end of the game, the threshold amount and the contents of the damage prevention account were communicated to the group. In the uncertainty treatments, the computer determined the exact threshold amount by drawing a random number from a uniform distribution either over the interval [0, 240] (uncertainty treatment), [84, 156] (warning-wide treatment) or [108, 132] (warning-narrow treatment). Once this information had been communicated to the group, participants completed a brief economic preferences questionnaire comprising single-item self-reported measures of risk aversion, loss aversion, trust, fairness, altruism and temporal discounting (see electronic supplementary material, statistical analyses). Participants were then paid in cash either the full remainder of their endowment (if the group contributions reached or exceeded the threshold amount) or 10% of the balance of their endowment (if the group failed to reach the threshold amount), in addition to a $10 attendance fee. The average payout was $20.15 (inclusive of attendance fee). The cash was concealed in envelopes to protect the anonymity of players.

## 3. Predictions and equilibria

### 3.1. Qualitative predictions

Consistent with earlier studies [3,11–13], we predicted that threshold uncertainty would undermine cooperation, such that group contributions and the probability of avoiding catastrophe would be reliably lower in the uncertainty treatment than in the certainty treatment. Based on the results of Barrett & Dannenberg [14], we further predicted that an early warning signal that reduced uncertainty to within 30% of the threshold value would fail to catalyse cooperation, such that group contributions and the probability of avoiding catastrophe would not differ between the uncertainty and warning-wide treatments, whereas an early warning signal that reduced uncertainty to within 10% of the threshold value would catalyse cooperation, such that group contributions and the probability of avoiding catastrophe would be higher in the warning-narrow than the uncertainty treatment.

### 3.2. Quantitative predictions

In addition to these empirically guided predictions, we also formulated a game-theoretic model of our experiment (see electronic supplementary material, analysis of experimental model). The imperfect information and repeated and multiple-player structure of the experiment allow for multiple Nash equilibria, and this complexity precludes a full equilibrium analysis. We therefore analyse the game under a set of simplifying assumptions, one of which is that all players are risk-neutral, and focus on two solutions—the internal cooperative equilibrium and Nash equilibrium. This is possible because the game has a single pay-off period at the end of the game and can therefore be partially analysed as an equivalent one-shot game. Barrett & Dannenberg [14] provide a similar analysis of such a game.

### 3.3. Equilibria

Table 2 presents the equilibrium predictions of our experimental model in terms of total contributions over all 10 rounds for the cooperative equilibrium (columns two and three) and the Nash equilibrium (columns four and five). The cooperative equilibrium is the best joint outcome for all group members. In the certainty treatment, this outcome arises when group members collectively contribute $120, and catastrophe is avoided with certainty. For the uncertainty treatment, it arises when group members collectively contribute $106.67,^1^ which is less than the expected value of the threshold ($120) and the upper limit of the threshold range ($240). These equilibria are an accurate guide to behaviour—our certainty and uncertainty treatments are equivalent to those used in the study by Dannenberg *et al.* [12], in which aggregate group contributions were €121.2 and €101.4, respectively. In the warning-wide and warning-narrow treatments, the cooperative equilibrium for the first five rounds is the same as for the uncertainty treatment, since these treatments are identical to one another up to this stage of the game. However, following the announcement of the revised threshold range at the start of round 6, the cooperative equilibrium for the warning-wide treatment increases to $156, whereas it increases to $132 for the warning-narrow treatment. Although collective pay-offs are maximized at these equilibria, the empirical studies reviewed suggest it is unlikely that group contributions will reach the upper bound in these treatments, especially in the warning-wide treatment.

**Table 2.** Cooperative and Nash equilibria for total contributions (columns two to five) as a function of treatment and expected contributions over rounds 1−5 (columns six and seven) and rounds 6−10 (columns eight and nine) to reach these equilibria.

	total				rounds 1−5		rounds 6−10	
treatment	cooperative		Nash		cooperative	Nash	cooperative	Nash
	rounds 1−10		rounds 1−10					
certainty	$120.00 (1.00)		$120.00 (1.00)		$60.00	$60.00	$60.00	$60.00
uncertainty	$106.67 (0.44)		$11.42 (0.05)		$53.34	$5.71	$53.34	$5.71
	rounds 1−5	rounds 6−10	rounds 1−5	rounds 6−10				
warning wide	$106.67 (0.44)	$156.00 (1.00)	$11.42 (0.05)	$99.42 (0.21)	$53.34	$5.71	$102.67	$93.71
warning narrow	$106.67 (0.44)	$132.00 (1.00)	$11.42 (0.05)	$124.71 (0.70)	$53.34	$5.71	$78.67	$119.00

The predictions based on cooperative equilibria are that catastrophe should be avoided with certainty in the certainty, warning-wide and warning-narrow treatments, whereas catastrophe should occur more often than not in the uncertainty treatment. These predictions are at variance with our empirically guided predictions.

The cooperative equilibrium does not take into account a player’s choice of strategy based on their beliefs about the actions of others. For this reason, a better guide to actual behaviour is likely to be provided by the Nash equilibrium, which refers to a set of player strategies in which each player has chosen their best response to the strategies they think their co-players will adopt. For the certainty treatment, the Nash equilibrium is $120 (contributing $0 is also a Nash equilibrium, albeit with a much lower payoff, making $120 the ‘focal’ contribution level [30]), which is the same as the cooperative equilibrium. For the uncertainty treatment, the Nash equilibrium is $11.42, which is considerably lower than the cooperative equilibrium and what we would expect based on actual behaviour [12]. In the warning-wide and warning-narrow treatments, the Nash equilibrium for the first five rounds is the same as for the uncertainty treatment. However, following the announcement of the revised threshold range at the start of round 6, the Nash equilibrium for the warning-wide treatment increases to $99.42, whereas it increases to $124.71 for the warning-narrow treatment. Both equilibria are less than the corresponding cooperative equilibria—much less in the case of the warning-wide treatment.

These predictions based on Nash equilibria are qualitatively consistent with our empirically guided predictions—catastrophe should be avoided with certainty in the certainty treatment and avoided more often than not in the warning-narrow treatment, whereas, in the uncertainty and warning-wide treatments, catastrophe should occur more often than not.

### 3.4. Contributions by stage of game

Table 2 breaks down these predictions according to how contributions to reach the cooperative and Nash equilibria should be divided over the first half (columns six and seven, respectively) and the second half (columns eight and nine, respectively) of the game. At the start of the game, groups in all treatments are led to expect that the information that they have been given regarding the location of the threshold is fixed and will not change during the course of the game. Therefore, we assume that at the outset groups will aim to contribute a total amount by the end of the game that will enable them to reach the cooperative or Nash equilibrium associated with the threshold information they have initially been given. Bearing in mind that our game-theoretic model treats our iterated game as a one-shot game and does not make predictions about contribution trajectories over rounds, we need to specify how groups should distribute their contributions over rounds. We adopt the simplifying assumption that players will contribute an equal and uniform amount over rounds to reach the cooperative or Nash equilibrium. Accordingly, the predictions for the certainty and uncertainty treatments are that groups should contribute half of the amount needed to reach these equilibria over rounds 1−5 and the remaining half over rounds 6−10. For the warning-wide and warning-narrow treatments, expected contributions to reach these equilibria in the first half of the game are calculated in the same way as for the uncertainty treatment and are equivalent, since before the mid-point of the game when the new threshold range is unexpectedly announced, these treatments are identical to one another. However, the announcement of the new threshold range in the warning-wide and warning-narrow treatments should prompt groups to adjust their contributions over the final five rounds towards a new equilibrium—specifically, the cooperative or Nash equilibrium associated with the newly announced threshold range. The expected contributions in these treatments following this announcement are the new cooperative and Nash equilibria, less the expected contributions in the first half of the game.

It should be noted that the abovementioned analysis is quite limited. Notably, the assumption that players will contribute equal and uniform amounts over rounds is unrealistic in most circumstances. Accordingly, the absolute quantities given in columns six to nine of table 2 are less important than the qualitative differences between treatments and stages of the game. In this regard, several qualitative trends are noteworthy. First, for contributions to reach both equilibria, a negative effect of threshold uncertainty is expected in both the first and second half of the game. The effect is larger in magnitude for contributions to reach the Nash equilibrium than to reach the cooperative equilibrium. Second, the effect of threshold uncertainty in the first half of the game should be the same for the three uncertainty treatments as they are identical to one another until the second half of the game. Third, an early warning signal should increase contributions in the second half of the game. For contributions towards reaching the cooperative equilibria, the effect of an early warning signal on cooperation levels is stronger in the warning-wide than the warning-narrow treatment, whereas the reverse is true for contributions towards reaching the Nash equilibria.

## 4. Results

The results are structured into four sections that examine the impact of the four experimental treatments on: (i) total contributions, (ii) contributions over rounds, (iii) the probability of avoiding catastrophe, and (iv) the link between proposals, pledges and contributions. For all analyses, the basic statistical unit is the group.

### 4.1. Total contributions

We begin by considering total group contributions across the four treatments and their relation to the cooperative and Nash equilibria (see columns two to five of table 2). Average group contributions collapsed over rounds (*M* ± s.d.) are markedly higher in the certainty ($119 ± 19.53) than the uncertainty treatment ($101.4 ± 22.21). Contributions in the certainty treatment are, on average, close to the cooperative and Nash equilibria (Wilcoxon = 36.00, *p* = 0.122), whereas contributions in the uncertainty treatment are close to the cooperative equilibrium (Wilcoxon = 21.00, *p* = 0.557) but significantly higher than the Nash equilibrium (Wilcoxon = 55.00, *p* = .002).^2^

Turning to the early warning treatments, average group contributions are marginally higher in the warning-wide ($109.4 ± 23.8) than the uncertainty treatment, whereas average group contributions are markedly higher in the warning-narrow ($124.2 ± 11.33) than the uncertainty treatment. Contributions in the warning-wide treatment are, on average, closest to the old cooperative equilibrium (Wilcoxon = 32.00, *p* = 0.695)—they are significantly higher than the old Nash equilibrium (Wilcoxon = 55.00, *p* = 0.002), significantly lower than the new cooperative equilibrium (Wilcoxon = 0.00, *p* = 0.002) and higher, albeit not significantly so (Wilcoxon = 41.00, *p* = 0.193), than the new Nash equilibrium. Contributions in the warning-narrow treatment are virtually identical to the new Nash equilibrium (Wilcoxon = 26.00, *p* = 0.922)—they are significantly higher than the old cooperative equilibrium (Wilcoxon = 55.00, *p* = 0.002) and Nash equilibrium (Wilcoxon = 55.00, *p* = 0.002), and lower, albeit not quite significantly so (Wilcoxon = 8.50, *p* = 0.059), than the new cooperative equilibrium.

In summary, total group contributions in the certainty treatment approximated the cooperative and Nash equilibria for this treatment, which are identical, whereas contributions in the uncertainty treatment approximated the cooperative equilibrium for this treatment. Total group contributions in the warning-wide treatment approximated the old cooperative equilibrium for this treatment, whereas contributions in the warning-narrow treatment approximated the new Nash equilibrium for this treatment.

### 4.2. Contributions over rounds

**Figure 2.**
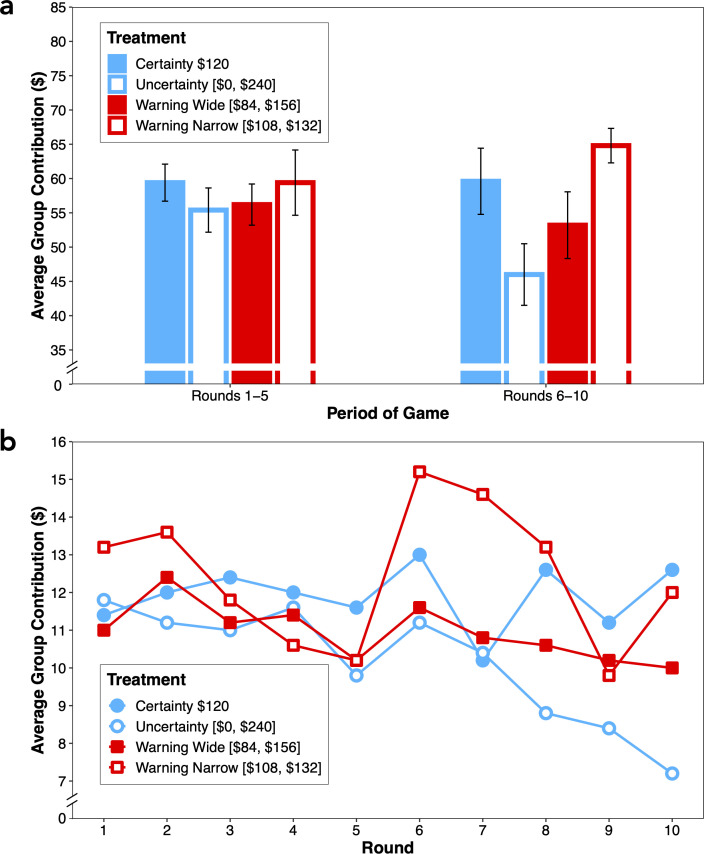
Contributions in the catastrophe avoidance game as a function of the four treatments. (a) Average group contributions in the first (rounds 1−5) and second (rounds 6−10) halves of the game (error bars represent standard errors). (b) Average group contributions as a function of each individual round of the game.

Next, we examine the pattern of contributions over the first and second halves of the game, which are plotted in figure 2*a*. These results can be contrasted with the expected contributions to reach the cooperative and Nash equilibria in table 2 for rounds 1−5 (columns six and seven, respectively) and rounds 6−10 (columns eight and nine, respectively). Qualitatively, the pattern of contributions over rounds 1−5 is most consistent with expected contributions to reach the cooperative equilibria. Numerically there is a small negative effect of threshold uncertainty for the uncertainty and warning-wide treatments, but contrary to those predictions, contributions in the warning-narrow treatment are equivalent to those in the certainty treatment. Notwithstanding the numerically lower contributions in the uncertainty and warning-wide treatments, contributions over rounds 1−5 do not differ significantly by treatment (Kruskal–Wallis, $\chi_{df=3}^{2}$ = 0.72, *p* = 0.869).

For contributions over rounds 6−10, the qualitative pattern is most consistent with expected contributions to reach the Nash equilibria. There is a pronounced effect of threshold uncertainty in the uncertainty treatment, but this effect is attenuated in the warning-wide treatment and eliminated in the warning-narrow treatment for which contributions are slightly higher than those in the certainty treatment. Accordingly, contributions over rounds 6−10 differ significantly by treatment (Kruskal–Wallis, $\chi_{df=3}^{2}$ = 10.95, *p* = 0.012). Contributions are significantly lower in the uncertainty than the certainty treatment (Mann–Whitney = 80.00, *p* = 0.025), confirming that threshold uncertainty reduced group contributions. Critically, while contributions do not differ significantly between the warning-wide and uncertainty treatments (Mann–Whitney = 33.500, *p* = 0.224), contributions are significantly higher in the warning-narrow than the uncertainty treatment (Mann–Whitney = 9.00, *p* = 0.002).

To scrutinize the data further, figure 2*b* plots the dynamics of group contributions over rounds for the four treatments. It can be seen that, with the exception of a trough in contributions in round 7, group contributions do not differ significantly over rounds in the certainty treatment (Freidman, $\chi_{df=9}^{2}$ = 7.89, *p* = 0.545), whereas group contributions decrease over rounds in the uncertainty treatment (Freidman, $\chi_{df=9}^{2}$ = 23.89, *p* = 0.004), with this decrease becoming more pronounced in the latter half of the game after the second set of proposals and pledges. Unlike the uncertainty treatment, group contributions in the warning-wide treatment did not tail off significantly over rounds (Freidman, $\chi_{df=9}^{2}$ = 5.90, *p* = 0.750), indicating that the early warning signal mid-game helped to stabilize group contributions. The pattern of group contributions in the warning-narrow treatment is uniquely different from the remaining treatments. Although group contributions decrease initially in the first half of the game, there is a punctuated peak in contributions in round 6 following the arrival of the early warning signal, after which contributions decay gradually, with a slight upturn in the final round (Freidman, $\chi_{df=9}^{2}$ = 15.61, *p* = 0.076).

In brief, while an early warning signal reducing uncertainty to within 30% of the threshold value did nothing to stimulate contributions, an early warning signal reducing uncertainty to within 10% of the threshold value increased contributions to a level comparable to that observed in the certainty treatment.

### 4.3. Probability of avoiding catastrophe

**Figure 3.**
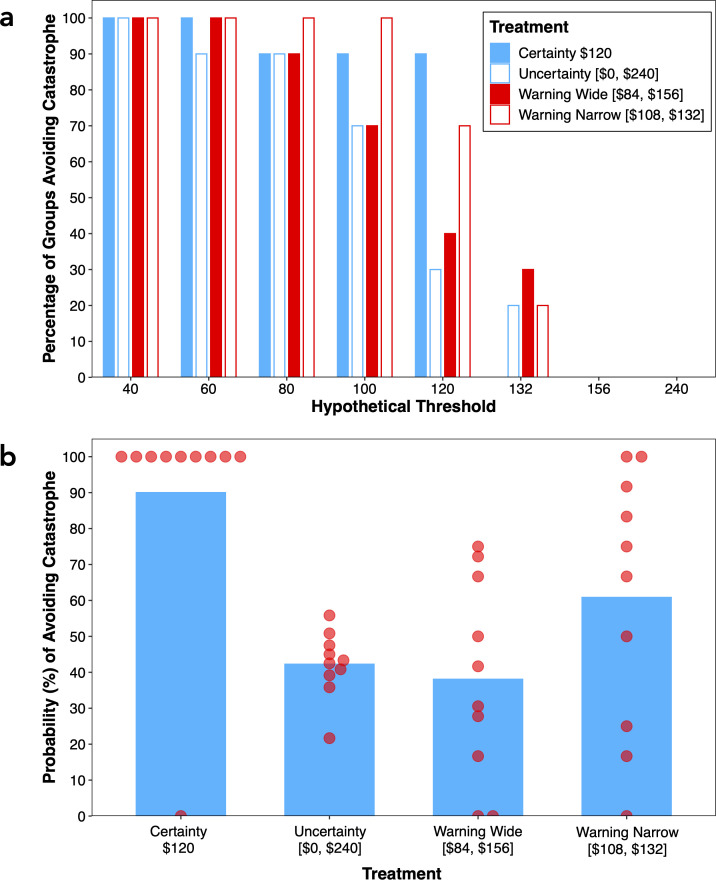
Probability of avoiding catastrophe as a function of the four treatments. (a) Percentage of groups avoiding catastrophe for various hypothetical threshold values. (b) Probability of avoiding catastrophe per group (denoted by the dots) and average catastrophe avoidance probability by treatment (denoted by the bars) after taking stochastic uncertainty into account.

We now examine the probability of avoiding catastrophe according to experimental treatment. The percentage of groups that would have averted catastrophe at various hypothetical thresholds is shown in figure 3*a*. At threshold values of $40, $60 and $80, most groups would have averted catastrophe, irrespective of treatment. At a threshold value of $100, 90% of groups in the certainty treatment, 70% of groups in the uncertainty and warning-wide treatments and 100% of groups in the warning-narrow treatment would have averted catastrophe.

Special attention must be given to the threshold value of $120 because it is the actual threshold value in the certainty treatment and the expected threshold value in the uncertainty treatments (uncertainty, warning wide and warning narrow). Thus, if we were to repeat the experiment many times, the average value of the threshold would be the expected value. Using the $120 threshold value, 90% of groups in the certainty treatment and 30% of groups in the uncertainty treatment would have averted catastrophe, a significant difference between treatments (Fisher exact, *p* = 0.020), confirming that threshold uncertainty reliably reduced the probability of group success. In the warning-wide treatment, 40% of groups would have averted catastrophe, which is not significantly higher than in the uncertainty treatment (Fisher exact, *p* = 1.000), indicating that an early warning signal that reduced uncertainty to within 30% of the threshold value did not increase the probability of group success. However, in the warning-narrow treatment, 70% of groups would have averted catastrophe, more than doubling the probability of group success compared to the uncertainty treatment, although this comparison did not reach statistical significance (Fisher exact, *p* = 0.179). It is likely that a higher number of observations would have revealed a significant difference between the two treatments.

Figure 3*a* shows group success rates at three additional hypothetical thresholds, namely $132, $156 and $240. These correspond to the upper threshold limits that groups must have reached in the warning-narrow, warning-wide and uncertainty treatments, respectively, to avert catastrophe with certainty. At $132, only 20% of groups in the uncertainty treatment, 30% of groups in the warning-wide treatment and 20% of groups in the warning-narrow treatment would have averted catastrophe. That more groups in the warning-narrow treatment did not reach the $132 threshold is noteworthy, given that a fair-share contribution of $22 per player would have ensured that catastrophe was averted with certainty. Unsurprisingly, at $156 and $240, none of the groups would have averted catastrophe.

A strength of the just presented analysis is that it compares the different treatments on a level playing field using a constant threshold for group success. However, a limitation is that, given a fixed contribution level, it does not factor into account variability in the odds of success across treatments based on the degree of uncertainty about the threshold (e.g. contributing $120 in the certainty treatment prevents catastrophe occurring with certainty, whereas in the uncertainty, warning-wide and warning-narrow treatments it still leaves a 50% chance of catastrophe occurring). Accordingly, we conducted a further analysis that took this stochastic uncertainty into account. Specifically, for each group, the probability, p, of avoiding catastrophe was determined by


(4.1) $$p\text{ =}\left\{ \begin{array}{ll} \text{0} & \text{if }Q_{T}<Q_{min}\\ \left(Q_{T}\text{-}Q_{min}\text{)/(}Q_{max}\text{-}Q_{min}\right) & \text{for }Q_{T}\in [Q_{min},Q_{max}\text{]}\\ \text{1} & \text{if }Q_{T}>Q_{max}, \end{array}\right.$$

where $Q_{T}$ is the total contribution, summed across the contributions of all six group members over all 10 rounds, and $Q_{min}$ and $Q_{max}$ are the lower and upper threshold limits, respectively, of the treatment to which the group belongs (for the warning-wide and warning-narrow treatments, these are the narrowed limits introduced mid-game).

The results are plotted in figure 3*b*, from which it can be seen that the probability of avoiding catastrophe differed appreciably across treatments (Kruskal–Wallis, $\chi_{df=3}^{2}$ = 14.97, *p* = 0.002). The probability was significantly higher in the certainty (90%) than in the uncertainty treatment (42%; Mann–Whitney = 90.00, *p* = 0.002), confirming that threshold uncertainty reduced the probability of group success. The probability of avoiding catastrophe was slightly lower in the warning-wide (38%) than in the uncertainty treatment, but not significantly so (Mann–Whitney = 44.00, *p* = 0.677), confirming that an early warning signal that reduced uncertainty to within 30% of the threshold value did not improve the odds of group success. Finally, the probability of avoiding catastrophe was higher in the warning-narrow (61%) than the uncertainty treatment—equivalent to a 45% increase in the probability of avoiding catastrophe—confirming that an early warning signal that reduced uncertainty to within 10% of the threshold value increased the probability of group success. However, the comparison only approached but did not reach statistical significance (Mann–Whitney = 69.00, *p* = 0.162). Once again, it is likely that the comparison would have attained statistical significance with a larger number of groups.^3^

### 4.4. Proposals, pledges and contributions

**Figure 4.**
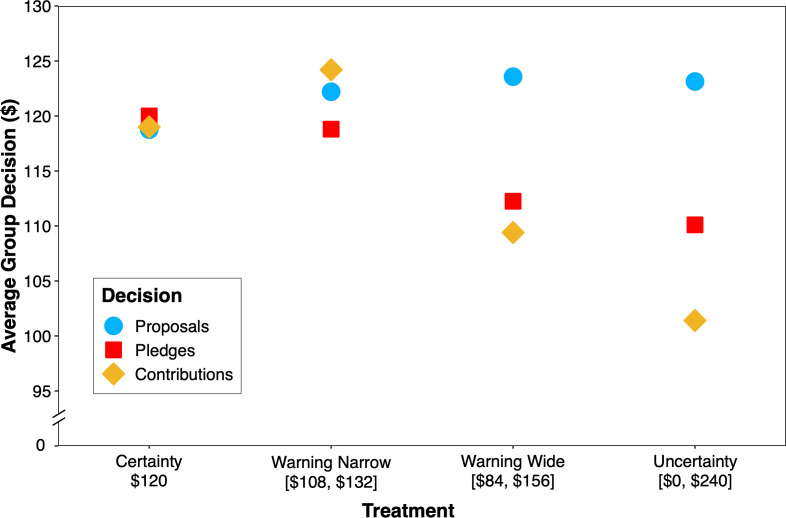
Average group proposals, pledges and contributions as a function of the four treatments.

Finally, we compared group proposals, pledges and contributions across treatments. Since group proposals and pledges in round 1 did not differ appreciably from those in round 6 (see electronic supplementary material, statistical analyses), for simplicity, we combined each into a single measure by averaging group proposals and pledges in the two rounds. The results are shown in figure 4, where the treatments have been organized, from left to right, in order of increasing threshold uncertainty (certainty < warning narrow < warning wide < uncertainty) instead of ascending treatment order. It can be seen that as threshold uncertainty increases, the gap between what groups propose to contribute, pledge to contribute and actually contribute also widens. In the certainty and warning-narrow treatments, group proposals, pledges and contributions fall closely in line. Indeed, in the warning-narrow treatment, contributions are numerically higher than proposals and pledges. By contrast, in the warning-wide and uncertainty treatments, pledges are less than proposals, and contributions, in turn, are less than pledges.

## 5. Discussion

Under conditions more reflective of the real game of climate change, the current study sought to replicate and extend the finding of Barrett & Dannenberg [14] that an early warning signal reducing threshold uncertainty to within 10% of the threshold value facilitates cooperation, whereas an early warning signal reducing threshold uncertainty by less than this amount has no effect on behaviour. To that end, we employed an iterated, rather than one-shot, catastrophe avoidance game in which threshold uncertainty was initially large in two treatments but subsequently reduced mid-game to within either 30% or 10% of the threshold value. We contrasted the behaviour of groups in these early warning treatments with that of groups in a certainty treatment, where the threshold was known with certainty, and an uncertainty treatment, where groups faced the same degree of threshold uncertainty throughout the game as that confronting groups initially in the early warning treatments.

### 5.1. Overview of key findings

Consistent with previous threshold experiments, using both one-shot [13,14] and iterated [11,12] games, we find that threshold uncertainty is a serious impediment to collective action. Compared to a certainty situation, threshold uncertainty reduced group contributions and increased the probability of catastrophe occurring. However, and critically, in line with [14] an early warning signal that reduced uncertainty to within 10% of the threshold value catalysed cooperation, increasing total group contributions to a level comparable to that witnessed under a certainty situation and reducing (albeit not quite reliably so) the probability of catastrophe occurring, compared to an uncertainty situation without a forewarning. By contrast, an early warning signal that reduced uncertainty to within 30% of the threshold value did little to stimulate group contributions. These results were obtained despite the shift from a one-shot to an iterated game, the use of dynamic rather than static thresholds in the early warning treatments and the fact that groups did not receive foreknowledge that the threshold uncertainty range would change mid-game. This confirms that the key results of Barrett & Dannenberg [14] are robust and not the consequence of specific features of their study methodology.

The novel contribution of our experiment—brought about by our use of an iterated game in which early warning signals arrived unexpectedly against the backdrop of large initial threshold uncertainty—lies in its demonstration that an early warning signal can move groups from one equilibrium to another, provided it reduces threshold uncertainty appreciably. Specifically, in the warning-narrow treatment, groups started by coordinating around the same cooperative equilibrium associated with the uncertainty treatment, but after the announcement of the early warning signal, they coordinated around the Nash equilibrium associated with the new threshold range. By contrast, in the warning-wide treatment, groups started by coordinating around the same cooperative equilibrium associated with the uncertainty treatment and continued to do so even after the announcement of the early warning signal. Previous iterated threshold experiments with a certain threshold have shown that coordination devices based on communication and intermediate thresholds can encourage groups to coordinate on a new equilibrium [24] or coordinate on one equilibrium and increase the probability of then coordinating on another [25]. Our results show that when the threshold is uncertain, a coordination device based on early warning signals can achieve similar results, provided that it reduces uncertainty to within a narrow range. This demonstration is important, we argue, because in the real game of climate change for an early warning signal to be effective, it would need to spur countries to coordinate on a different equilibrium to that which they are currently rallying around. Our study suggests that this is possible provided an early warning signal is sufficiently accurate in pinpointing where a climate tipping point is located.

Although our results are largely consistent with those of Barrett & Dannenberg [14], along with the results of Dannenberg *et al*. [12] they suggest that the effect of threshold uncertainty, while robust, is not as strong in an iterated game as in a one-shot game. Using equation (4.1) to compute catastrophe avoidance probabilities, in Barrett & Dannenberg [13,14], the probability of avoiding catastrophe is 85% in the certainty treatment and ≈ 0% in the 100−200 treatment where threshold uncertainty is at its widest. In our study, the probability of avoiding catastrophe is 90% in the certainty treatment and 42% in the uncertainty treatment. The corresponding values for Dannenberg *et al*. [12] are comparable: 100% versus ≈ 42%, respectively. This result is noteworthy given that in our study, and that of Dannenberg *et al*. [12], the threshold uncertainty range is larger than in Barrett & Dannenberg [13,14], which might lead one to expect that the impact of threshold uncertainty would be larger, not smaller, in magnitude.

Although the handicap of threshold uncertainty is not as pronounced in our study as in Barrett & Dannenberg [13,14], somewhat counterintuitively, so too is the impact of an early warning signal on cooperation. In our study, an early warning signal that reduced uncertainty to within 10% of the threshold value increased the probability of avoiding catastrophe from 42% to 61%, compared to 90% in the certainty treatment. By contrast, in Barrett & Dannenberg [14], it increased the probability of avoiding catastrophe from 0% to 75%, compared to 85% in the certainty treatment. However, the threshold uncertainty range in our warning-narrow treatment was wider than in the 145−155 treatment of Barrett & Dannenberg [14], which may explain why our early warning signal was less effective at catalysing cooperation—in absolute terms, the reduction in threshold uncertainty was greater in their study than in ours. Moreover, in our study, the reduction in uncertainty occurs as a surprise mid-game rather than being known throughout their one-shot game, which may render it harder to avoid the threshold.

These nuanced differences between studies should be interpreted with some caution, as the studies differ along dimensions other than those discussed above. Indeed, what is most impressive is the remarkable degree of correspondence between our results and those of Barrett & Dannenberg [14], notwithstanding their methodological differences. Our findings agree with theirs in demonstrating that threshold uncertainty is a handicap to cooperation and that for an early warning signal to spur cooperation, it must reduce uncertainty to within a narrow range.

### 5.2. Implications for climate negotiations

If a red line for dangerous climate change could be identified, fear of crossing it would spur collective action to avoid it. Accordingly, a key role of science in climate politics is to identify tipping points that can facilitate global cooperation [31]. The science of early warning signals offers the tantalizing prospect that uncertainty about the location of a climate tipping point may be reduced as we get closer to it. Our results and those of Barrett & Dannenberg [14] cannot directly address the question of how accurately we would need to know where a climate tipping point lies to trigger collective action to avoid it. However, the two sets of results suggest that uncertainty may need to be reduced to somewhere between 30 and 10% of the threshold value. It is worrying, therefore, that there are question marks regarding whether an early warning signal could provide the level of precision necessary in these studies to transform the collective action problem [32].

Even if such a level of precision is possible, our results suggest that an early warning signal offers no assurance that the threshold will be avoided. A worrying aspect of our findings is that groups do not adhere to the precautionary principle of risk management [33]. In our warning-narrow treatment, groups must contribute an amount equal to or greater than $132, the upper threshold limit, to avert catastrophe with certainty. Group contributions in this treatment, on average, were just above the expected threshold value of $120, which requires a fair-share contribution of $20 per group member. Increasing this contribution by a mere $2 per group member would be sufficient to avoid catastrophe with certainty. Yet, only 20% of groups in this treatment did so. Indeed, our groups were contented to contribute $120, as reflected in their aggregate proposals, despite the fact this still leaves a 50% chance of catastrophe occurring. In terms of actual group contributions, rather than proposals, there remains a residual 39% chance of catastrophe occurring in this treatment.

There are other limitations of early warning signals. The best way to reduce uncertainty about a threshold is to get closer to it, but by then, it may already be too late to take emergency measures to avoid crossing it. There is also the risk that an early warning signal may go undetected, meaning we may not know about the location of the threshold until it has already been breached. Continued investment in the identification and detection of early warning signals is evidently warranted, as our results attest, and even if they arrive too late to mobilize collective action to avoid climate tipping points, they may nevertheless serve as an aid to pre-emptive adaptation [22]. It is clear, though, that early warning signals do not constitute a silver bullet, and climate negotiators will therefore need to entertain other strategies to cultivate the cooperation needed to avoid a climate catastrophe.

As noted by Barrett & Dannenberg [15], the problem with contemporary climate agreements is that it is Mother Nature, rather than the countries themselves, that provides the enforcement. That is, it is Mother Nature’s threat to tip the climate system into chaos if a climate tipping point is breached that provides the incentive for collective action. However, threshold uncertainty undermines the credibility of this threat. Since uncertainty about climate thresholds is difficult to reduce, enforcement is out of the control of the countries—it is Mother Nature that holds all the cards. As Barrett & Dannenberg [15] note, if Mother Nature cannot provide the enforcement, then countries must do so themselves.

One way to think about this challenge is in terms of the game-theoretic model of threshold uncertainty developed by Barrett [34]. According to this model, there exists a theoretical dividing line in threshold uncertainty. To the right of this dividing line, when threshold uncertainty is large, the climate cooperation problem is a prisoner’s dilemma, whereas to the left of the dividing line, when threshold uncertainty is small, the climate cooperation problem is a coordination game. Cooperation is difficult to achieve in the prisoner’s dilemma because there is only one Nash equilibrium, and it is a non-cooperative equilibrium in which all countries defect. By contrast, cooperation is easier to achieve in the coordination game because there are two Nash equilibria, a dangerous equilibrium in which all countries defect and a safe equilibrium in which all countries cooperate. The safe equilibrium is ‘focal’ [30] or psychologically prominent since no country wants to suffer catastrophe. Cooperation, thus, simply requires that countries coordinate on the mutually preferred safe equilibrium.

Viewed through this lens, the challenge for climate negotiators is to devise strategic enforcement mechanisms that allow countries to escape the prisoner’s dilemma by converting it into a coordination game. An example of the use of strategic enforcement is the Montreal Protocol on Substances that Deplete the Ozone Layer, one of the most effective international environmental agreements ever negotiated. The success of this agreement lies in its strategic use of the threat to restrict trade in controlled substances between parties and non-parties [35,36], which converts the ozone depletion prisoner’s dilemma into a coordination game [37]. One way to achieve this same transformation to tackle the climate problem is by linking trade agreements with climate protection and using the strategic threat to impose tariffs on countries that do not take appropriate measures to reduce their emissions to enforce climate cooperation [38].

### 5.3. Potential limitations and future directions

There are some potential limitations of our study that merit comment. First, the initial threshold uncertainty in the uncertainty treatments ($0–$240)—which ranged from group members not needing to contribute anything to their entire endowment to avert catastrophe—is much larger than the threshold uncertainty (1.5−2°C) in the real game of climate change. An early warning signal that reduces uncertainty to within 10% of the threshold value might be more effective at catalysing cooperation when the initial threshold uncertainty is smaller, as it must surely be in the real climate game. Thus, our study may have underestimated the potential effectiveness of early warning signals. However, it is non-trivial to translate the threshold uncertainty in the real climate game into proportional uncertainty, as represented in our experiment.

Second, the early warning signals in our study arrived unexpectedly. Arguably, it would have been more reflective of the real game of climate change to have forewarned groups at the outset regarding the prospect of a change in the degree of uncertainty about the threshold mid-game. This is because ever since the climate negotiations in Cancun [39], countries have been alert to the possibility that they may need to limit warming to 1.5°C, rather than 2°C. Indeed, a special report by the IPCC [40] highlighted the pressing need to restrict warming to 1.5°C—this call to action served as an early warning of the need for more stringent climate action. Foreknowledge of the prospect of an early warning signal could enhance the effectiveness of such signals, but it could also undermine them by, for example, promoting undue optimism or wishful thinking [41,42]. Only further experiments comparing the impact of early warning signals with and without foreknowledge of their possible arrival will answer this question.

Third, we only examined the consequences for cooperation of early warning signals in which the expected value of the threshold remained the same, but the uncertainty around it was reduced. However, an early warning signal could also signify a shift in the expected value of the threshold, indicating that it is closer than originally anticipated, thus requiring emergency action to avoid it. Such a shift might be expected to cause groups to choke under pressure; alternatively, it might provide the sense of urgency required to catalyse groups into action. Once again, only further experiments can elucidate which of these possibilities is most likely.

## 6. Conclusions

Uncertainty about the threshold for dangerous climate change renders it difficult to mobilize collective action to avoid it. Our research and that of Barrett & Dannenberg [14] demonstrates that early warning signals of an approaching tipping point can catalyse cooperation to prevent it from being exceeded, but only when such signals reduce uncertainty to within a very narrow range. Even then, our research implies that we cannot be assured countries will adhere to the precautionary principle and do what it takes to avoid the threshold with certainty. There remain important gaps in our knowledge of early warning signals that must be filled, such as how the prospects of cooperation are affected by early warning signals that indicate a shift in the expected value of the threshold, not merely a narrowing of the threshold range. However, the limitations of this approach mean climate negotiators must consider alternative strategies to motivate collective action other than the fear of crossing a dangerous threshold. Rather than leaving enforcement in the hands of Mother Nature, a better approach may be for climate negotiators to wrestle back control over the enforcement problem by using strategic treaty design to transform the climate change prisoner’s dilemma into a coordination game, thus recreating the conditions that exist when the threshold is certain.

## Data Availability

Data and relevant code for this research work are stored in GitHub: https://github.com/mark-hurlstone/Hurlstone-White-Newell-2024-Supplementary-Resources.git and have been archived within the Zenodo repository [43]. Supplementary material is available online [44].

## Footnotes

^1^
Readers may wonder why the cooperative equilibrium is not centred at the expected value of the threshold as in other work [29]. This is because when the threshold is exceeded, it is assumed that a proportion of the pay-off is lost (90%) rather than all of it. The initial endowment is also important in terms of determining the optimal contribution.

^2^
We note that the accuracy of the Nash equilibrium contribution prediction for the uncertainty treatment could probably be improved by rerunning the analysis for an ‘average’ level of risk aversion. Most players will want a lower level of risk than the ‘representative’ risk-neutral player in our simplified experimental model.

^3^
We note that the analyses of the probability of avoiding catastrophe are less sensitive than the analyses of group contributions, and a power analysis suggests that we are statistically somewhat underpowered to detect what is a modest-sized effect (i.e. the uncertainty versus warning-narrow comparison). Nevertheless, our sample size of 10 groups per treatment is consistent with sample-size norms for research in this field [9]. Accordingly, our power to detect a reliable difference is no less than that of other studies in the literature. In presenting formal analyses of these data, we have gone beyond convention in the field—most authors only report these data visually but do not subject them to statistical analysis [12–14], instead limiting inferential statistics to comparisons based on contribution levels.
